# Surveillance and Analysis of Avian Influenza Viruses, Australia

**DOI:** 10.3201/eid1612.100776

**Published:** 2010-12

**Authors:** Philip M. Hansbro, Simone Warner, John P. Tracey, K. Edla Arzey, Paul Selleck, Kim O’Riley, Emma L. Beckett, Chris Bunn, Peter D. Kirkland, Dhanasekaran Vijaykrishna, Bjorn Olsen, Aeron C. Hurt

**Affiliations:** Author affiliations: The University of Newcastle, Newcastle, New South Wales, Australia (P.M. Hansbro, E.L. Beckett);; Department of Primary Industries, Attwood, Victoria, Australia (S. Warner, K. O’Riley);; Orange Agricultural Institute, Orange, New South Wales, Australia (J.P. Tracey);; Elizabeth Macarthur Agriculture Institute, Menangle, New South Wales, Australia (K.E. Arzey, P.D. Kirkland);; Australian Animal Health Laboratories, Geelong, Victoria, Australia (P. Selleck);; Department of Agriculture, Fisheries and Forestry, Canberra, Australian Capital Territory, Australia (C. Bunn);; Duke-NUS Graduate Medical School, Singapore (D. Vijaykrishna);; Kalmar University, Kalmar, Sweden (B. Olsen);; Uppsala University, Uppsala, Sweden (B. Olsen);; World Health Organization Collaborating Centre for Reference and Research on Influenza, Melbourne, Victoria, Australia (A.C. Hurt)

**Keywords:** Viruses, avian influenza virus, ecology, lineage, phylogenetics, shorebird, surveillance, waterfowl, Australia, research

## Abstract

We investigated carriage of avian influenza viruses by wild birds in Australia, 2005–2008, to assess the risks to poultry industries and human health. We collected 21,858 (7,357 cloacal, 14,501 fecal) samples and detected 300 viruses, representing a detection rate of ≈1.4%. Rates were highest in autumn (March–May) and differed substantially between bird types, areas, and years. We typed 107 avian influenza viruses and identified 19 H5, 8 H7, and 16 H9 (40% of typed viruses). All were of low pathogenicity. These viruses formed clearly different phylogenetic clades to lineages from Eurasia or North America, suggesting the potential existence of Australian lineages. H7 viruses were similar to highly pathogenic H7 strains that caused outbreaks in poultry in Australia. Several periods of increased detection rates (numbers or subtypes of viruses) were identified. This study demonstrates the need for ongoing surveillance to detect emerging pathogenic strains and facilitate prevention of outbreaks.

## Abstract 2

TOC Summary: A lineage unique to Australia has been identified.

Shorebirds (Charadriiforme*s*) and wild waterfowl (Anseriformes) represent the major natural reservoirs of avian influenza viruses (AIVs). These birds can carry all 16 hemagglutinin (HA) and 9 neuraminidase (NA) subtypes (*1*); the viruses typically cause asymptomatic infections in these hosts. Studies in Europe and North America demonstrated the following: AIV carriage is highest in autumn but may also be high in spring; prevalence among shorebirds and ducks is increased during their northward and southward migrations, respectively; and distribution, prevalence, and subtypes involved vary from year to year (*2**,**3*). Interspecies transmission of AIV in several species of wild birds has been documented; however, the most frequent adaptation of these viruses occurs in domestic gallinaceous poultry.

The respiratory tract of poultry and gastrointestinal tract of waterfowl are replication sites for AIVs, and poultry are incubators for the progression of low-pathogenicity avian influenza (LPAI) virus into highly pathogenic avian influenza (HPAI) virus (*4**–**6*), usually through the acquisition of polybasic amino acids at the HA cleavage site. HPAI, particularly HPAI (H5N1), may induce up to 100% deaths in poultry and cause substantial economic losses (*4**,**7**–**9*). Strains that are highly pathogenic in gallinaceous species may cause a range of clinical signs in other avian species, from mild illness to highly contagious and fatal disease. H5 and H7 AIVs have the propensity to become HPAI and thus are a significant risk to the poultry industry. These subtypes and H9 have also caused disease and death in humans. Subtype H5N1 first caused outbreaks in wild migratory waterfowl in the People’s Republic of China in 2002 and in domestic poultry in Hong Kong Special Administrative Region, China, in 2003 (*10*). The World Health Organization has since confirmed 433 human cases of avian influenza (H5N1) with 262 deaths (*11*).

AIVs may be transported by infected migratory birds (*12**–**14*). Shorebirds and waterfowl usually survive infection, and transmission by migratory waterfowl over long distances within Asia and between continents has been documented (*15**–**17*). Nevertheless, the role of migratory birds in the distribution and transmission of AIVs remains controversial. Managing the potential threat of transport of AIVs by wild birds requires appropriate surveillance programs that assess the occurrence, subtypes, and pathogenicity of isolates that the birds carry.

Australia is isolated by sea, and shorebirds make up the majority of long-distance migratory birds that visit the continent (3 million/year [*12*]). These birds breed in Siberia (May–July) and stop off throughout Asia (April–May, July–September) in areas where HPAI (H5N1) epizootics have recently occurred (e.g., Vietnam, Thailand, Hong Kong, China, Indonesia) (*18*). Most arrive in Australia in spring (August–September) and depart in autumn (March). Shorebirds are known to carry a variety of AIVs, including subtype H5N1 (*1*). Wild waterfowl, such as ducks, geese and swans are common in Australia. However, they do not migrate out of Australia in large numbers, although they do undertake intracontinental movements and occupy the same habitats as migratory shorebirds. Collectively, these factors provide an environment that allows the assessment of the import of AIVs by migratory birds and transmission to, and distribution by, local waterfowl.

Until recently, only small and historical studies of AIVs have been undertaken in Australia (*14**,**19**–**22*). No outbreaks of HPAI H5 viruses have been identified, despite the close proximity of Indonesia, where AIV (H5N1) is endemic and outbreaks frequently occur. Five outbreaks of HPAI have occurred in Australia; all outbreaks were caused by H7 viruses. In all cases of disease, transmission of LPAI H7 from wild birds and subsequent mutation to HPAI after serial passage in chickens was considered the probable source (*13**,**23**,**24*). Nevertheless, the source of infection in wild birds has not been identified. Therefore, surveillance for AIVs is needed in Australia in localities where large numbers of migratory shorebirds and waterfowl occur in close proximity to poultry operations (*13*). We examined the occurrence and subtypes of AIVs carried by migratory shorebirds and waterfowl in southeast Australia over a 4-year period.

## Methods

### Sample Collection

**Figure 1:**
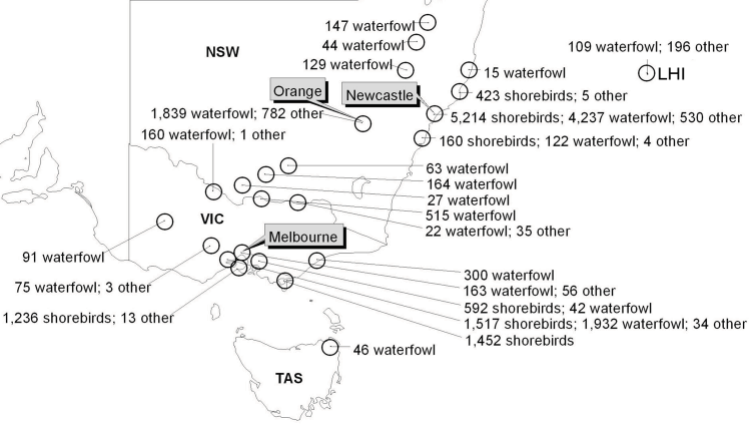
Sampling sites for avian influenza in Australia. Most avian fecal and cloacal samples were collected from wetlands in coastal and inland New South Wales (NSW) or around Melbourne, Victoria (VIC), with minor sampling sites around Old Bar, Sydney, and Albury, NSW; Lord Howe Island (LHI); and northeastern Tasmania (TAS). Shorebirds refers to migratory shorebirds only.

Sampling site selection (2005–2008) was based on abundance of migratory shorebirds, risk for transmission to waterfowl inhabiting the same area, proximity of commercial poultry, and human population density. Areas around Newcastle and Orange, New South Wales (*13*), and Melbourne, Victoria, were selected. Samples were also collected opportunistically from other locations (Figure 1). Sites were generally inland swamps or coastal wetlands.

Most samples were collected from coastal wetlands or inland swamps around Newcastle and Orange, New South Wales, and Melbourne, Victoria, with small numbers from other sites (Figure 1). Samples from Tasmania were included with samples from Victoria for analysis. Coastal New South Wales and Victoria samples were from sites co-inhabited by large numbers of migratory shorebirds and waterfowl. A total of 21,858 samples were collected and tested during 2005–2008 (Table 1). Of these, 10,003 were from migratory shorebirds, 10,231 from waterfowl, and 1,624 from other bird species. Samples from other bird species were from birds trapped incidentally or were collected opportunistically. In most instances, the species, or pairs of sister species (e.g., grey/chestnut teal, bar-tailed/black-tailed godwit), that produced the feces collected were known. Identification of species sampled was identified by observing the bird, the bird’s footprints, and the size and shape of feces.

**Table 1:** Summary of numbers and bird types sampled and PCR-positive rates of avian influenza viruses detected, by month and state, New South Wales and Victoria, Australia, 2005–2008*

Bird type and month	New South Wales				Victoria				Total		
	No. samples†	Pos	PPR, %		No. samples†	Pos	PPR, %		No. samples†	Pos	PPR, %
Shorebirds											
Jan	704 (5)	1	0.14		206 (206)				910	1	0.11
Feb	1,087 (8)	2	0.18		901 (901)	7	0.78		1,988	9	0.45
Mar	939 (2)	2	0.21						939	2	0.21
Apr	328	6	1.80						328	6	1.80
May	163	5	3.10						163	5	3.10
Jun	217	4	1.80						217	4	1.80
Jul	101	1	0.99						101	1	0.99
Aug	126								126		
Sep	529	1	0.19						529	1	0.19
Oct	586				404 (404)	7	1.70		990	7	0.71
Nov	694 (7)	10	0.76		627 (627)				1,321	10	0.76
Dec	323				2,068 (2,068)	5	0.24		2,391	5	0.21
Total	5,797	32	0.55		4,206	19	0.45		10,003	51	0.51
Waterfowl											
Jan	265	4	1.50		406	23	5.70		671	27	4.00
Feb	393	9	2.30		265	2	0.75		658	11	1.70
Mar	591 (51)	14	2.40		836 (836)	44	5.30		1,427	58	4.10
Apr	836 (80)	25	3.00		241	5	2.10		1,077	30	2.80
May	1,262 (232)	18	1.40		175	10	5.70		1,437	28	1.90
Jun	740 (241)	10	1.40		14				754	10	1.30
Jul	525 (127)	13	2.50		4				529	13	2.50
Aug	496 (69)	10	2.00						496	10	2.00
Sep	596 (111)	25	4.20		5	1	20.0		601	26	4.30
Oct	381 (97)	2	0.50		329	9	2.70		710	11	1.50
Nov	770 (513)	6	0.80		366	6	1.60		1,136	12	1.00
Dec	578 (89)	10	1.70		157	1	0.64		735	11	1.50
Total	7,433	146	2.00		2,798	101	3.60		10,231	247	2.40
Other											
Jan	62 (12)				1 (1)				63		
Feb	198 (1)				10 (10)				208		
Mar	35 (27)				1 (1)				36		
Apr	314 (12)	1	0.40						314	1	0.30
May	159 (60)								159		
Jun	72 (44)								72		
Jul	17 (6)				3 (3)				20		
Aug	107 (28)								107		
Sep	133 (118)								133		
Oct	196 (149)				1 (1)				197		
Nov	259 (154)				23 (23)				282		
Dec					33 (33)	1	3.00		33	1	3.00
Total	1,552	1			72	1	1.40		1,624	2	0.10
Total for all birds	14,782	182	1.32%		7,076	121	1.70		21,858	300	1.40

*Shorebirds refers to migratory shorebirds only. Sampling periods were defined as calendar months. Pos, number of PCR-positive results; PPR, PCR-positive rate. †Numbers in parenthesis indicate the numbers of cloacal samples; the remainder were fecal samples.

**Figure 2:**
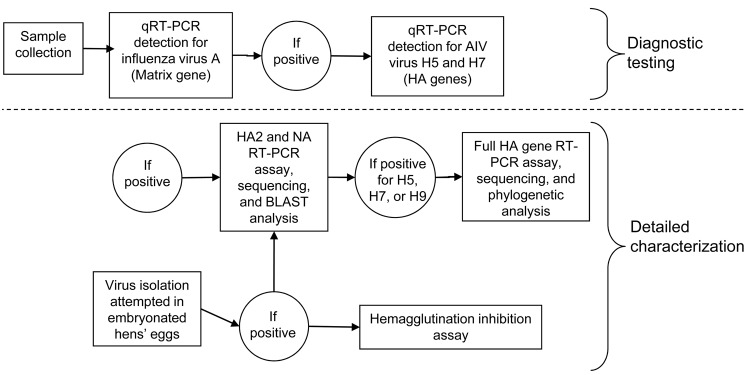
Procedures followed in avian influence surveillance and analysis, Australia, 2005–2009. qRT-PCR, real-time quantitative reverse transcription–PCR; AIV, avian influenza virus; HA, hemagglutinin; NA, neuraminidase; BLAST, BLAST analysis (http://blast.ncbi.nlm.nih.gov).

Samples were fresh feces or cloacal swabs (*14*). Fecal samples were collected from roosting or feeding flocks, and the species involved was recorded. Cloacal samples were collected from birds captured by cannon netting, funnel traps, and hand-held nets, and from ducks shot for recreation, damage mitigation, or conservation. Samples were placed in phosphate-buffered gelatin saline or brain-heart-infusion broth base, each containing penicillin (2 × 10^6^ IU/L), streptomycin (0.2 mg/mL), gentamicin (0.5 mg/mL), and amphotericin B (500 U/mL), and transported chilled to our laboratories at the University of Newcastle, the Department of Primary Industries or the Orange Agricultural Institute for storage at –80°C until analysis (Figure 2).

### PCR Detection of AIVs

Viral RNA was extracted according to manufacturers’ instructions by using MagMax96 viral RNA (Life Technologies, Scoresby, Victoria, Australia), or RNeasy isolation kits (QIAGEN, Doncaster, Victoria, Australia). AIVs were detected by real-time quantitative reverse transcription–PCR (qRT-PCR) by using the conserved matrix gene as the amplification target (*25**,**26*). Influenza A–positive samples were tested by using specific primers targeting H5 and H7 subtypes (*26*). Proportion tests (Pearson χ^2^ statistics in R [*27*]) were used to test differences in influenza A–positive by PCR rates according to season.

### AIV Subtype Determination

To determine AIV subtypes, HA2 and NA genes were amplified by conventional PCR and sequenced (*14*). Sequences were compared with known sequences by BLAST search (www.ncbi.nlm.nih.gov/genomes/FLU/FLU.html) to determine subtype and relatedness to other viruses. For H5, H7, and H9 subtypes, the full HA genes were sequenced and HA cleavage sites were assessed to determine potential pathogenicity (*14*).

### Phylogenetic Analysis

**Figure 3:**
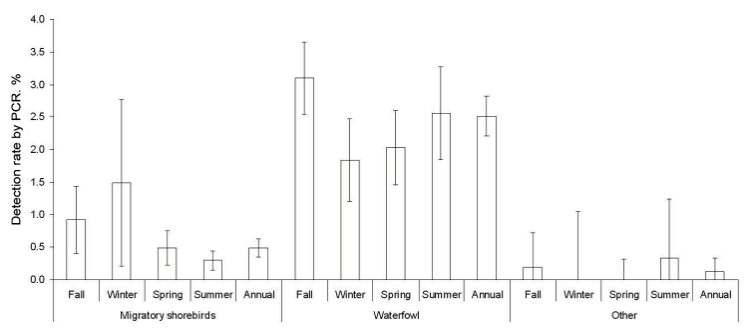
Phylogenetic analysis of avian influenza viruses from Australia. Viruses were subtyped, and hemagglutinin genes from subtypes H5 (A), H7 (B), and H9 (C) were sequenced (**boldface**) and compared with isolates from other geographic locations. Only bootstrap values >50 are shown. Scale bar indicates nucleotide substitutions per site. All sequences were submitted to GenBank (accession numbers pending). LPAI, low-pathogenicity avian influenza; HPAI, highly pathogenic avian influenza.

Phylogenetic trees were constructed for H5, H7, and H9 viruses by comparison of the relatedness of the subtypes isolated in this study with those from other geographic locations (Figure 3). HA genes of 10 H5, 3 H7, and 8 H9 viruses from this study were compared with those of representative subtypes of major AIV lineages from GenBank. Sequences were assembled and edited with SeqMan, DNASTAR Lasergene 8. Geneious (Biomatters Ltd, Auckland, New Zealand) and Se-Al (http://evolve.zoo.ox.ac.uk/) were used for alignment. MRMODELTEST 2.2 (www.abc.se/~nylander/) was used to determine the appropriate DNA substitution model and γ-rate heterogeneity. The best-fit model was used to generate neighbor-joining trees by using PAUP* 4.0 (*28*). Only strains from which a full HA gene sequence was obtained were included. Estimates of phylogenies were calculated from 1,000 neighbor-joining bootstrap replicates.

## Results

### AIVs Detected

Three hundred AIVs were detected by qRT-PCR, representing a total PCR-positive detection rate of 1.4% ,of which 51 (17%) were detected in migratory shorebirds (including 16 bar-tailed godwits, 14 red-necked stints, 11 eastern curlews, and 7 red knots) and 247 in waterfowl (including 224 dabbling ducks), corresponding to rates of 0.51% and 2.4%, respectively (Table 1). Two viruses were detected in other birds (Eurasian coot and whiskered tern). *Numenius* spp. waders (predominantly eastern curlew, 11/690, 1.6%) were the most common shorebird carriers. Dabbling ducks had slightly higher detection rates (224/7,607, 2.9%) compared with all waterfowl.

### PCR-positive Samples

**Figure 4:**
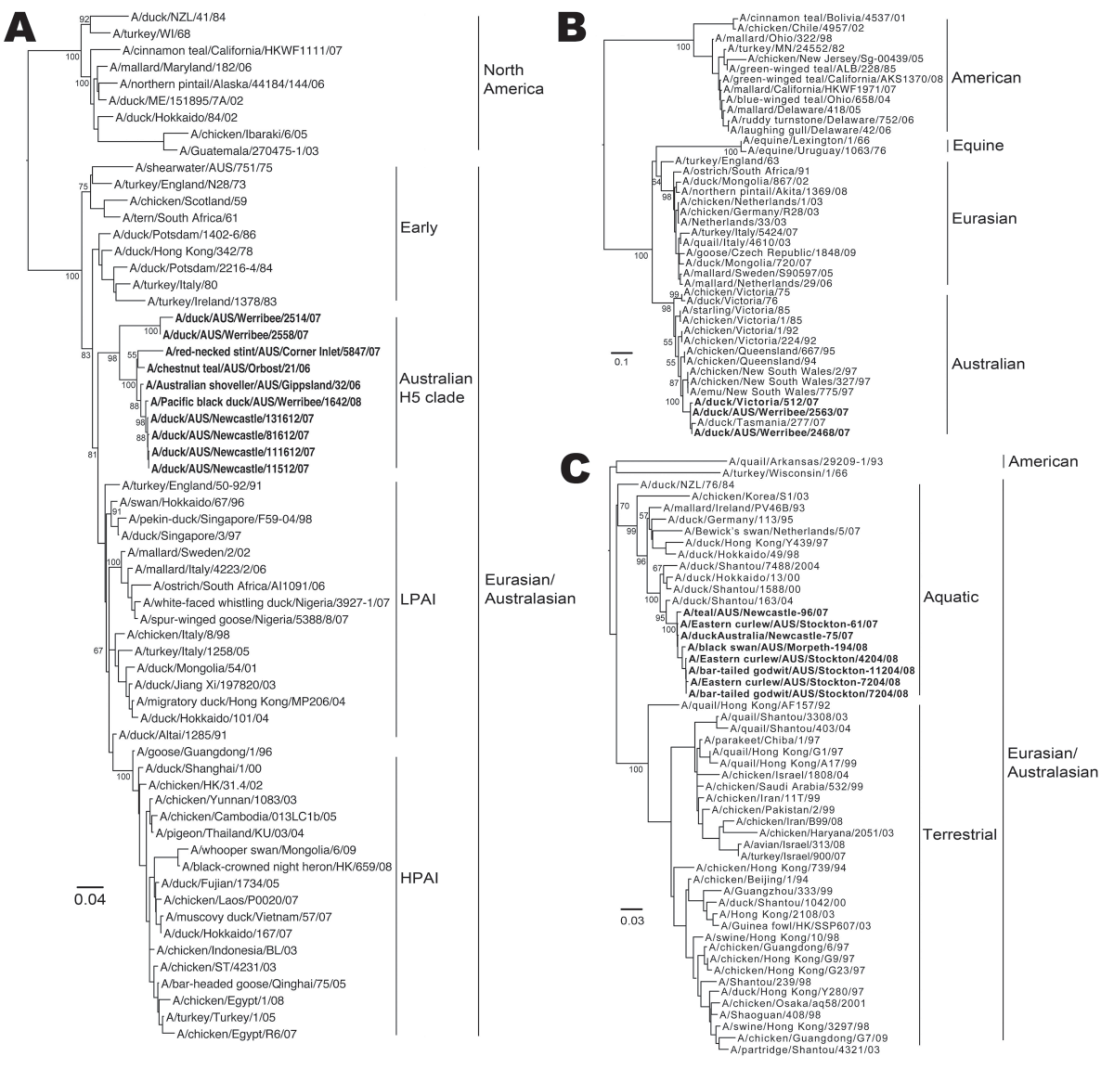
PCR-positive rates of avian influenza virus by season and species. Error bars indicate 95% confidence intervals.

PCR-positive detection rates were similar for migratory shorebirds (0.55% vs. 0.45%) and waterfowl (2.0% vs. 3.6%) between New South Wales and Victoria (Table 1). Rates were highest in autumn and early winter (April–June, χ^2^ = 18.0, degrees of freedom [df] = 3, p = 0.0004) in migratory shorebirds, and in autumn (April–May, χ^2^ = 11.2, df = 3, p = 0.01) in waterfowl (Figure 4). Rates were similar for different years: migratory shorebirds 0.65% in 2005, 0.50% in 2006, 0.46% in 2007, and 0.72% in 2008; and waterfowl 2.7% in 2006, 2.6% in 2007, and 3.7% in 2008. However, rates differed substantially for different bird types, areas, and years, which could explain the high variability observed in seasonal trends (Figure 4). For example, the rate for migratory shorebirds in coastal New South Wales in 2008 (0.72%, mostly bar-tailed godwit and eastern curlew) was double that in Victoria in 2006 (0.38%), rates for waterfowl in Victoria (4.8%, mostly in Pacific black duck) in 2008 were almost double those in 2007 (2.8%), and rates for dabbling ducks in Victoria in 2008 (6.3%) were 3-fold greater than in coastal New South Wales in 2006 (1.9%). Rates were generally similar for different years for migratory shorebirds sampled in New South Wales and Victoria; however, rates reached 5.2% for waterfowl (mostly grey and chestnut teal) in March 2006 in Victoria, compared with 3.6% at other times.

### AIV Ecology

It was possible to subtype 107/300 (36%) AIVs detected by qRT-PCR (Table 2; Technical AppendixTables 1–3). It was not possible to subtype all AIVs because the conventional PCR used for subtyping was not as sensitive as the surveillance qRT-PCR. Notably, 19 H5, 8 H7, and 16 H9 AIVs were identified. No H5 or H7 AIVs contained multiple basic cleavage sites, a known molecular determinant for HPAI; therefore, all were classified as LPAI. H5, H7, and H9 subtypes represented a high proportion (43/107, 40%) of all viruses subtyped. H9 subtypes were the most common viruses identified in migratory shorebirds (5/11, 45%). H3 and H5 viruses were the most common subtypes identified in waterfowl (21/96, 22%, and 18/96, 19%, respectively). One H5 and 5 H9 AIVs were detected in migratory shorebirds, 1 H9 was from a black swan, and the remainder (18 H5, 8 H7, and 10 H9) were from dabbling ducks. In PCR-positive samples for which NA subtype was determined, we detected 1 N1, 4 N3, 1 N5, 5 N6, 4 N7, and 1 N9 (Technical Appendix Table 1 ). The NAs associated with H5, H7, and H9 viruses were of the following subtypes: H5N3 (1), H5N7 (2), H7N1 (1), H7N6 (2), and H7N7 (1).

**Table 2:** Hemagglutinin subtypes of avian influenza viruses detected, by month and state, New South Wales and Victoria, Australia, 2005–2008

Species and month	New South Wales	Victoria	Total
Shorebirds*			
Feb		1 H5	1 H5
Apr	4 H9		4 H9
May	2 H3, 1 H9		2 H3, 1 H9
Nov	1 H12	1 H6	1 H6, 1 H12
Dec		1 H4	1 H4
Total	2 H3, 5 H9, 1 H12	1 H4, 1 H5, 1 H6	2 H3, 1 H4, 1 H5, 1 H6, 5 H9, 1 H12
Waterfowl			
Jan	1 H5	1 H2, 2 H4, 2 H5, 2 H7, 1 H8	1 H2, 2 H4, 3 H5, 2 H7, 1 H8
Feb	1 H5, 3 H7		1 H5, 3 H7
Mar	1 H3, 2 H9, 1 H12	3 H3, 5 H5, 1 H11, 2 H12	4 H3, 5 H5, 2 H9, 1 H11, 3 H12
Apr	5 H3, 1 H4, 1 H6, 5 H9		5 H3, 1 H4, 1 H6, 5 H9
May	1 H3, 1 H5, 1 H6, 4 H9, 2 H11	2 H11	1 H3, 1 H5, 1 H6, 4 H9, 4 H11
Jun	1 H1, 2 H4, 1 H5,		1 H1,2 H4, 1 H5
Jul	3 H1, 1 H4, 3 H8, 1 H11, 1 H12		3 H1,1 H4, 3 H8, 1 H11, 1 H12
Aug	3 H2, 3 H3		3 H2, 3 H3
Sep	5 H3, 1 H4, 1 H5, 1 H7, 3 H8, 1 H10		5 H3, 1 H4, 1 H5, 1 H7, 3 H8, 1 H10
Oct		1 H3, 1 H8	1 H3, 1 H8
Nov	1 H3	2 H7	1 H3, 2 H7
Dec	1 H3 6 H5, 1 H8	1 H1	1 H1, 1 H3, 6 H5, 1 H8
Total	4 H1, 3 H2, 17 H3, 5 H4, 11 H5, 2 H6, 4 H7, 7 H8, 11xH9, 1 H10, 3 H11, 2 H12	1 H1, 1 H2, 4 H3, 2 H4, 7 H5, 4 H7, 2 H8, 3 H11, 2 H12	5 H1, 4 H2, 21 H3, 7 H4, 18 H5, 2 H6, 8 H7, 9 H8, 11 H9, 1 H10, 6 H11, 4 H12
Total for all birds	4 H1, 3 H2, 19 H3, 5 H4, 11 H5, 2 H6, 4 H7, 7 H8, 16 H9, 1 H10, 3 H11, 3 H12	1 H1, 1 H2, 4 H3, 3 H4, 8 H5, 1 H6, 4 H7, 2 H8, 3 H11, 2 H12	5 H1, 4 H2, 23 H3, 8 H4, 19 H5, 3 H6, 8 H7, 9 H8, 16 H9, 1 H10, 6 H11, 5 H12

*Shorebirds refers to migratory shorebirds only.

The detection of PCR-positive samples was sporadic and was increased in some periods, particularly in ducks, in which larger numbers of AIVs were identified at the same time and location (Technical Appendix Table 4 ). During periods of increased detection, rates of up to 6.2% and 12.3% were found for migratory shorebirds and waterfowl, respectively. These events occurred throughout the year but were more common in autumn (March–May) and early spring (September). Some evidence showed different seasonal increases in rates for particular subtypes of AIVs. Twenty of 23 H3 subtypes were primarily detected from autumn to early spring (March–September), whereas 16/19 H5 and 7/8 H7 subtypes were detected from late spring to early autumn (November–March), and all 16 H9 subtypes were detected in autumn (March–May). Most H3 (13/23), H5 (12/19), and H7 (5/8) strains were detected in 2007, whereas half (8/16) of the H9 strains were from 2007 and half were from 2008. Notably, 8 H5 viruses were identified in summer (December–February) in New South Wales in 2007–2008, and only 1 strain of a different subtype (H4) was identified during this period. Also, notable increases in detection rates of H9 subtypes occurred in New South Wales in autumn in 2007 (April–May) and 2008 (March–April). In addition, rates involving numerous different subtypes increased on 3 occasions: 2 H3, 3 H5, 1 H11, 1 H12, from dabbling ducks, Victoria, March 2006; 1 H2, 1 H4, 2 H5, 2 H7, 1 H8, from ducks, Victoria, January 2007, and 3 H2, 6 H3, 1 H4, 1 H5, 1 H10, mostly from teal, New South Wales, August–September 2007. However, these increases may be the result of the large numbers of samples collected on these dates (365, 341, and 504, respectively).

Increased detection rates of individual AIV subtypes were generally localized because the same subtypes were only identified at the same time from different sites on 3 occasions (Technical Appendix Table 1 ): 1 H12, New South Wales, and 1 H12, Victoria, in March 2006; 2 H9, coastal New South Wales, and 3 H9 inland in May 2007; and 2 H11, New South Wales, and 2 H11, Victoria, in May 2008. Some evidence for cross-species infection was found with the same subtypes of virus identified in different species at the same time and location: 2 H9 in both bar-tailed godwits and eastern curlews in April 2008 in New South Wales. Full HA sequences for the 4 strains demonstrated >99.6% nt similarity; the 2 strains from the bar-tailed godwits showed 100% nt homology (Figure 3). Limited evidence was shown for the cross-species infection of migratory shorebirds and waterfowl, with the same subtype isolated from each group on just 2 occasions, both involving H9 viruses in New South Wales: 1 H9 from an eastern curlew and 1 H9 from a duck in May 2007 and H9s from 2 bar-tailed godwits, 2 eastern curlews, 1 black swan, and 1 chestnut teal, all in April 2008. The H9 strains from the bar-tailed godwit and eastern curlew had a >98.8% nt similarity to the H9 strain from the black swan (Figure 4). Sequence data were not available for the duck or chestnut teal AIVs.

### Phylogenetic Analysis

All H5 viruses detected in this study clustered closely together and were clearly divergent from other LPAI H5 viruses from Eurasia and North America (Figure 3, panel A). Both the Australian and the Eurasian lineages appear to have evolved from an early lineage of H5 viruses that includes a range of strains from 1959 through 1986. The H7 strains identified in this study have a close genetic relationship with HPAI H7 viruses previously isolated in Australia during 1975–1997 and as a group are clearly distinct from Eurasian and North American H7 lineages (Figure 3, panel B). Notably, all Australian H7 viruses were closely related to the strains that caused pathogenic outbreaks in poultry in Australia and thus may identify a potential environmental source of these viruses. Eurasian H9 strains have evolved into 2 discrete lineages that are carried by aquatic or terrestrial birds (Figure 4, panel C). The Australian H9 strains detected in this study again grouped closely and, as a lineage, diverged from the Asian aquatic H9 viruses but were distinct from Eurasian and North American lineages. Although the Australian H9 viruses were a less discrete lineage than H5 and H7 viruses, the maximum bootstrapping value (100) confirmed that they formed their own distinct lineage. Taken together, these results indicate that the viruses within each subtype in Australia are closely related and form Australian-specific lineages that are distinct from other lineages.

## Discussion

This large surveillance effort for AIVs in Australia longitudinally and geographically characterized the extent and profile of AIVs in wild birds. We detected 300 AIVs from ≈22,000 samples tested and subtyped 107 of these. *Anas* species ducks were the predominant carriers, and the peak of detection occurred in autumn. Detection rates varied among different locations and times. Numerous H5, H7, and H9 AIVs were detected, although no HPAI strains were identified. The Australian viruses within each subtype were closely related and formed separate clades from Eurasian or North American lineages, indicating that separate lineages of H5, H7, and H9 AIVs are circulating in Australia.

### PCR-positive Rates

Most AIV ecology studies have been conducted in Europe and the United States (*2*). In Australia, the overall PCR-positive detection rate of 1.4% for all bird species is similar. However, rates of 0.51% for shorebirds and 2.4% for waterfowl are similar to, or less than, those detected in other geographic regions where 0.2%–20% of shorebirds and 7%–37% of waterfowl were carriers (*2**,**29**–**32*). Our results agree with those of historical Australian studies in which rates of 0.6% for all birds and 1%–5% for ducks were found (*19**–**21*). Rates for AIVs in shorebirds in Australia were previously unknown. We found rates were highest for dabbling ducks, which is consistent with findings of other studies (*2**,**29*). These higher rates may be a result of the ducks’ feeding technique of filtering soft mud, which may be an environment conducive to the persistence of AIVs.

### Autumn and Winter Detection Rates

Detection rates for migratory shorebirds were highest during April–June and were highest in overwintering eastern curlews. This finding contrasts with results of studies from North America that show a low prevalence in winter (*29*). The Australian winter is considerably milder than winter in areas studied in North America, and differences among winter rates may result from these climatic differences.

Shorebirds migrate to Australia down the East Asian–Australian flyway and then subsequently disperse throughout Australia. Our results suggest that migratory shorebirds are not commonly carrying AIVs into Australia, which would be indicated by peak detections in newly arrived birds in September, but that they become infected during autumn and winter in Australia. This provides further evidence that AIV infection is not maintained during migration (*33*), although studies in Europe have shown that ducks can carry AIVs during migration (*2*).

Rates in waterfowl were high in early spring (September), which corresponds with the period when young birds arrive in coastal Australia (*34*). This finding agrees with those of studies from other locations, which show that immunologically naive juvenile birds carry more viruses, which may have been transmitted from adult birds or the environment (*29**,**32*).

### Variability and Increases in Detection Rates

AIV detection rates were variable and seasonal, and periods of increased rates occurred. Large numbers of viruses were detected during some sampling periods but not others, and some subtypes were often identified at the same time and location. Detection of H5 viruses increased in the summer of 2007–08 in New South Wales; all H9 viruses were detected in New South Wales, and most H5, H7, and H9 viruses were identified in different years. These increases in detection rates were generally localized to particular times and places. These results are supported by findings of our smaller previous study that found that subtypes H11N9 and H4N8 were common in migratory shorebirds in November 2004 but not since then (*14*). Detection of the same subtypes at the same times indicated limited evidence of cross-infection, which suggests that occasionally viruses may be passed between bird species (e.g., shorebird to shorebird) and families (e.g., between shorebirds and waterfowl).

### Phylogenetics

In this study, H1–H12 and all NA subtypes, excluding N2, N4, and N8, were detected, a similar level of diversity as that observed in other studies (*2*). The AIVs of particular concern are H5, H7, and H9 because they have been associated with outbreaks in poultry and disease in humans. Notably, in this study, we found that these were the most common subtypes, representing 40% of all AIVs identified; however, no HPAI (H5N1) strains were detected. This pattern is different from that observed in other locations, e.g., H4, H6, and H7 were most common in Sweden, and H1, H2, H4, and H6 dominated in Germany and North America (*3**,**5*). This difference may indicate variations in host–virus interactions in Australia. Phylogenetic analysis showed that AIVs of the same subtype detected in Australia are closely related and are distinct from viruses isolated from other geographic locations. We attempted to isolate viruses from on all PCR-positive samples, but only 3 viruses were recovered (2 H7, 1 H5, all from fecal samples). Antigenic hemagglutination inhibition assays (*35*) of the 2 H7 viruses showed they were antigenically similar to 5 HPAI H7 strains that caused outbreaks in poultry in Australia during 1976–1992 but were not similar to 2 Asian H7 viruses (data not shown). The genetic and limited antigenic data demonstrate that little genetic evolutionary change has occurred and suggest that no antigenic change has occurred in Australian H7 viruses over >30 years.

Previous studies of AIVs have shown that globally 2 separate HA AIV lineages occur: Eurasian and North American (*2*). However, our study provides clear evidence that Australian AIVs, rather than being part of the Eurasian lineage, have diverged and may be considered as belonging to a different lineage. The genetically discrete Australian lineage suggests that endemic circulation and evolutionary isolation of strains in Australia have occurred and provides little evidence for the importation of exotic strains by migratory birds (*33*).

This and other studies highlight the need for continued longitudinal surveillance, particularly in areas with large numbers of migratory birds and waterfowl located close to commercial poultry, and in northern Australia, which is nearest to areas where HPAI (H5N1) is endemic. Further genetic and antigenic characterization of AIVs in Australian wild bird populations should be performed. Surveillance programs can identify peaks in occurrence and may act as an early warning system. Such measures are essential for maximizing biosecurity for the poultry industry and public health agencies.

## Supplementary Material

Technical Appendix Details of individual avian influenza viruses detected, numbers of individual avian influenza virus hemagglutinin subtypes detected, and summary of periods (month and year) of increased detection, and seasonal occurrence of selected hemagglutinin avian influenza virus.
