# Supplementary material for: Surveillance and Analysis of Avian Influenza Viruses, Australia
Source: Emerg Infect Dis. 2010 Dec;16(12):1896–904. doi: 10.3201/eid1612.100776 (PMC3294589; doi:10.3201/eid1612.100776)
Supplement: Technical Appendix — Details of individual avian influenza viruses detected, numbers of individual avian influenza virus hemagglutinin subtypes detected, and summary of periods (month and year) of increased detection, and seasonal occurrence of selected hemagglutinin avian influenza virus. [file 10-0776-Techapp_5p.pdf]

Article DOI: 10.3201/eid1612.100776

Surveillance and Analysis of Avian Influenza Viruses, Australia

Technical Appendix

Technical Appendix Table 1. Details of individual avian influenza viruses detected, Australia, 2005–2008*

	Date	Location	Area	Subtype
Migratory shorebirds				
A/Red-necked Stint/Australia/Gippsland/1001/2005	2005 Nov 6	Gippsland	Vic	H6
A/Black-tailed Godwit/Australia/Stockton/19/2007	2006 Nov 25	Stockton	NSW	H12N5
A/Red-necked Stint/Australia/Corner Inlet/2847/2007	2007 Feb 15	Corner Inlet	Vic	H5N7
A/Eastern Curlew/Australia/Stockton/61/2007	2007 May 2	Stockton	NSW	H9
A/Eastern Curlew/Australia/Stockton/214212/2007	2007 May 3	Stockton	NSW	H3
A/Eastern Curlew/Australia/Stockton/214213/2007	2007 May 3	Stockton	NSW	H3
A/Red-necked Stint/Australia/Werribee/1001/2007	2007 Dec 28	Werribee	Vic	H4
A/Bar-tailed Godwit /Australia/Stockton/7204/2008	2008 Apr 20	Stockton	NSW	H9
A/Bar-tailed Godwit/Australia/Stockton/11204/2008	2008 Apr 20	Stockton	NSW	H9
A/Far Eastern Curlew/Australia/Stockton/4204/2008	2008 Apr 20	Stockton	NSW	H9
A/Far Eastern Curlew/Australia/Stockton/7204/2008	2008 Apr 20	Stockton	NSW	H9
Waterfowl				
A/Grey Teal/Australia/Tocumwal/E4/2005	2005 Nov 4	Tocumwal	NSW	H3N3
A/Grey Teal/Australia/Tocumwal/N53/2005	2005 Dec 15	Tocumwal	NSW	H3
A/Grey Teal/Australia/Tasmania/19/2006	2006 Mar 11	NE TAS	Vic	H11N9
A/Chestnut Teal/Australia/Tasmania/21/2006	2006 Mar 11	NE TAS	Vic	H5
A/Pacific Black Duck/Australia/Pitt Town/254/2006	2006 Mar 17	Pitt Town	NSW	H12
A/Australian Shoveller/Australia/Gippsland/32/2006	2006 Mar 18	Orbost	Vic	H5N3
A/Chestnut Teal/Australia/Gippsland/11/2006	2006 Mar 18	Gippsland	Vic	H12
A/Chestnut Teal/Australia/Gippsland/35/2006	2006 Mar 18	Gippsland	Vic	H3
A/Chestnut Teal/Australia/Orbost/21/2006	2006 Mar 18	Orbost	Vic	H5
A/Chestnut Teal/Australia/Barinsdale/35/2006	2006 Mar 18	Bairnsdale	Vic	H3
A/Duck/Australia/Newcastle/436/2006	2006 Apr 4	Kooragang Island	NSW	H6
A/Pacific Black Duck/Australia/Jerilderie/AD9/2006	2006 May 9	Jerilderie	NSW	H6
A/Grey Teal/Australia/Morundah/AD35/2006	2006 May 10	Morundah	NSW	H3
A/Pacific Black Duck/Australia/Orange/AK32/2006	2006 Jun 26	Orange	NSW	H4
A/Pacific Black Duck/Australia/Orange/AK37/2006	2006 Jun 29	Orange	NSW	H4N6
A/Pacific Black Duck/Australia/AL44/2006	2006 Jun 29	Orange	NSW	H1
A/Pacific Black Duck/Australia/Orange/AN41/2006	2006 Jun 30	Orange	NSW	H5N3
A/Pacific Black Duck/Australia/Orange/AN57/2006	2006 Jul 5	Orange	NSW	H1
A/Pacific Black Duck/Australia/Orange/AN58/2006	2006 Jul 5	Orange	NSW	H1
A/Wood Duck/Australia/Orange/AN59/2006	2006 Jul 5	Orange	NSW	H1
A/Pacific Black Duck/Australia/Orange/AO16/2006	2006 Jul 6	Orange	NSW	H8
A/Pacific Black Duck/Australia/Orange/AO18/2006	2006 Jul 6	Orange	NSW	H8
A/Grey Teal/Australia/Orange/AO17/2006	2006 Jul 6	Orange	NSW	H8
A/Duck/Australia/Newcastle/490/2006	2006 Jul 22	Lenaghan's Fort	NSW	H4N6
A/Pacific Black Duck/Australia/Jerilderie/O14/2006	2006 Jul 25	Jerilderie	NSW	H12
A/Pacific Black Duck/Australia/Jerilderie/O26/2006	2006 Jul 25	Jerilderie	NSW	H11
A/Pacific Black Duck/Australia/Caroona/AD9/2006	2006 Aug 22	Jerilderie	NSW	H3
A/Grey Teal/Australia/Inverell/AB39/2006	2006 Sep 24	Inverell	NSW	H8
A/Duck/Australia/Morpeth/2696/2006	2006 Sep 26	Morpeth	NSW	H3N7
A/Grey Teal/Australia/Jerilderie/AR25/2006	2006 Sep 29	Jerilderie	NSW	H7N7
A/Grey Teal/Australia/Jerilderie/AR20/2006	2006 Sep 29	Jerilderie	NSW	H8
A/Pacific Black Duck/Australia/Jerilderie/AR32/2006	2006 Sep 29	Jerilderie	NSW	H8
A/Grey Teal/Australia/Werribee/490/2006	2006 Nov 24	Werribee	Vic	H7N6
A/Grey Teal/Australia/Werribee/512/2006	2006 Nov 24	Werribee	Vic	H7N6
A Teal/Australia/Morpeth/21421/2006	2006 Dec 4	Morpeth	NSW	H8
A/Duck/Australia/Werribee/1866/2007	2006 Dec 27	Werribee	Vic	H1
A/Duck/Australia/Werribee/2536/2007	2007 Jan 10	Werribee	Vic	H7

A/Duck/Australia/Werribee/2558/2007	2007 Jan 10	Werribee	Vic	H5N7
A/Duck/Australia/Werribee/2566/2007	2007 Jan 10	Werribee	Vic	H4N6
A/Duck//Australia/Werribee/2468/2007	2007 Jan 10	Werribee	Vic	H7N1
A/Duck/Australia/Werribee/2514/2007	2007 Jan 10	Werribee	Vic	H5
A/Duck/Australia/Werribee/2750/2007	2007 Jan 14	Werribee	Vic	H8
A/Duck/Australia/Werribee/2757/2007	2007 Jan 14	Werribee	Vic	H2
A/Duck/Australia//Newcastle/21426/2007	2007 Feb 7	Kooragang Island	NSW	H7
A/Duck/Australia//Newcastle/21427/2007	2007 Feb 7	Kooragang Island	NSW	H7
A/Duck/Australia/Newcastle/21428/2007	2007 Feb 7	Kooragang Island	NSW	H7
A/Duck/Australia/Werribee/3148/2007	2007 Mar 19	Werribee	Vic	H12
A/Duck/Australia/Newcastle/74/2007	2007 Apr 3	Kooragang Island	NSW	H3
A/Duck/Australia/Newcastle/75/2007	2007 Apr 3	Kooragang Island	NSW	H9
A/Duck/Australia/Newcastle/80/2007	2007 Apr 3	Kooragang Island	NSW	H3
A/Teal/Australia/Newcastle/84/2007	2007 Apr 3	Kooragang Island	NSW	H3
A/Teal/Australia/Newcastle/96/2007	2007 Apr 3	Kooragang Island	NSW	H9
A/Teal/Australia/Newcastle/4540/2007	2007 Apr 5	Deep Pond	NSW	H3
A/Teal/Australia/Newcastle/211004/2007	2007 Apr 10	Deep Pond	NSW	H9
A/Pacific Black Duck/Australia/Orange/BL46/2007	2007 May 3	Orange	NSW	H9
A/Pacific Black Duck/Australia/Orange/BL48/2007	2007 May 3	Orange	NSW	H9
A/Duck/Australia/Morpeth/112/2007	2007 May 6	Morpeth	NSW	H9
A/Pacific Black Duck/Australia/Orange/BY16/2007	2007 May 30	Orange	NSW	H9
A/Pacific Black Duck/Australia/Orange/BY22/2007	2007 May 30	Orange	NSW	H5
A/Teal/Australia/Morpeth/21782007	2007 Aug 17	Morpeth	NSW	H3
A/Teal/Australia/Morpeth/2188/2007	2007 Aug 17	Morpeth	NSW	H2
A/Teal/Australia/Morpeth/8188/2007	2007 Aug 17	Morpeth	NSW	H3
A/Teal/Australia/Morpeth/11188/2007	2007 Aug 17	Morpeth	NSW	H2
A/Teal/Australia/Morpeth/12188/2007	2007 Aug 18	Morpeth	NSW	H2
Teal/Australia/Morpeth/469/2007	2007 Sep 6	Morpeth	NSW	H10
A/Teal/Australia/Morpeth/1069/2007	2007 Sep 6	Morpeth	NSW	H3
A/Teal/Australia/Morpeth/1469/2007	2007 Sep 6	Morpeth	NSW	H3
A/Teal/Australia/Morpeth/1569/2007	2007 Sep 6	Morpeth	NSW	H4
A/Teal/Australia/Morpeth/1869/2007	2007 Sep 6	Morpeth	NSW	H3
A/Duck/Australia/Morpeth/179/2007	2007 Sep 7	Morpeth	NSW	H5
A/Duck/Australia/Morpeth/1079/2007	2007 Sep 7	Morpeth	NSW	H3
A/Duck/Australia/Werribee/2007	2007 Oct 18	Werribee	Vic	H3
A/Black Swan/Australia/Werribee/2007	2007 Oct 18	Werribee	Vic	H8
A/Duck/Australia/Kooragang/11512/2007	2007 Dec 15	Kooragang Island	NSW	H5
A/Duck/Australia/Kooragang/71612/2007	2007 Dec 16	Kooragang Island	NSW	H5
A/Duck/Australia/Kooragang/81612/2007	2007 Dec 16	Kooragang Island	NSW	H5
A/Duck/Australia/Kooragang/111612/2007	2007 Dec 16	Kooragang Island	NSW	H5
A/Duck/Australia/Kooragang/131612/2007	2007 Dec 16	Kooragang Island	NSW	H5
A/Duck/Australia/Kooragang/161612/2007	2007 Dec 16	Kooragang Island	NSW	H5
A/Chestnut Teal/Australia/Kooragang/3141/2008	2008 Jan 14	Kooragang Island	NSW	H5
A/Duck/Australia/Werribee/1361/2008	2008 Jan 23	Werribee	Vic	H4
A/Duck/Australia/Kooragang/4202/2008	2008 Feb 20	Kooragang Island	NSW	H5
A/Duck/Australia/Morpeth/5133/2008	2008 Mar 13	Morpeth	NSW	H9
A/Pacific Black Duck/Australia/Morpeth/2143/2008	2008 Mar 14	Morpeth	NSW	H3
A/Pacific Black Duck/Australia/Morpeth/10143/2008	2008 Mar 14	Morpeth	NSW	H9
A/Pacific Black Duck/Australia/Werribee/1391/2008	2008 Mar 31	Werribee	Vic	H3
A/Pacific Black Duck/Australia/Werribee/1453/2008	2008 Mar 31	Werribee	Vic	H5
A/Pacific Black Duck/Australia/Werribee/1462/2008	2008 Mar 31	Werribee	Vic	H5
A/Black Swan/Australia/Morpeth/194/2008	2008 Apr 9	Morpeth	NSW	H9
A/Chestnut Teal/Australia/Morpeth/1594/2008	2008 Apr 9	Morpeth	NSW	H3
A/Chestnut Teal/Australia/Morpeth/5154/2008	2008 Apr 15	Morpeth	NSW	H9
A/Duck/Australia/Morpeth/8194/2008	2008 Apr 19	Morpeth	NSW	H4
A/Pacific Black Duck/Australia/Werribee/1663/2008	2008 May 9	Werribee	Vic	H11
A/Pacific Black Duck/Australia/Werribee/1673/2008	2008 May 14	Werribee	Vic	H11
A/Chestnut Teal/Australia/Morpeth/31165/2008	2008 May 16	Morpeth	NSW	H11
A/Chestnut Teal/Australia/Morpeth/35165/2008	2008 May 16	Morpeth	NSW	H11

***** Conventional bird species names are used (Christidis L, Boles W. Systematics and Taxonomy of Australian Birds: CSIRO; 2008).
NSW, New South Wales; Vic, Victoria; NE TAS, northeastern Tasmania.

Technical Appendix Table Table 2 Numbers of individual avian influenza virus hemagglutinin subtypes detected per state, Australia, 2005–2008

Subtype	Total no.	New South Wales	Victoria
H1	5	4	1
H2	4	3	1
H3	23	19	4
H4	8	5	3
H5	19	11	8
H6	3	2	1
H7	8	4	4
H8	9	7	2
H9	16	16	
H10	1	1	
H11	6	3	3
H12	5	3	2

Technical Appendix Table 3. Numbers of individual avian influenza virus hemagglutinin subtypes detected in migratory shorebirds and waterfowl per year, Australia, 2005–2008

Migratory shorebirds				
	2005	2006	2007	2008
	No., subtype	No., subtype	No., subtype	No., subtype
			2 H3	
			1 H4	
			1 H5	
			5 H9	4 H9
	1 H6			
		1 H12		
Waterfowl				
		5 H1		
			4H2	
	2H3	5 H3	11 H3	3 H3
		3 H4	2 H4	2 H4
		4 H5	11 H5	3 H5
		2 H6		
		3 H7	5 H7	
		7 H8	2 H8	
			7 H9	
			1 H10	
		2 H11		4 H11
		3 H12	1 H12	

Technical Appendix Table 4. Summary of periods (month and year) of increased detection, and seasonal occurrence of selected hemagglutinin avian influenza virus*

New South Wales						Victoria					
Species name	Year, mo,	No.	No. Pos	PPR, %	No. Subtype	Species name	Year, mo	No.	No. Pos	PPR, %	No. subtype
Non-subtyped											
Shorebirds											
Bar-tailed Godwit	2008 Apr	64	4	6.2		Red Knot	2005 Oct	144	4	2.8	
Eastern Curlew	2007 May	109	4	3.7							
Eastern Curlew	2007 Jun	92	3	3.3							
Bar-tailed Godwit	2006 Nov	238	9	3.8							
Waterfowl											
Duck sp.	2007 Feb	70	4	5.7		Duck Sp.	2007 Jan	341	20	5.6	
Duck sp.	2008 Feb	114	5	4.4		Aust. Shoveler	2006 Mar	148	8	5.4	
Grey Teal	2006 Mar	18	1	5.6		Chestnut Teal	2006 Mar	204	18	8.8	
Duck sp.	2008 Mar	114	6	5.3		Pacific Black Duck	2006 Mar	94	6	6.4	
Duck sp.	2007 Apr	146	11	7.5		Pacific Black Duck	2008 Apr	105	5	4.8	
Grey Teal	2006 May	17	1	5.9		Pacific Black Duck	2008 May	103	10	9.7	
Pacific Black Duck	2006 Jun	88	6	6.8		Duck Sp.	2006 Oct	88	4	4.5	
Pacific Black Duck	2006 Jul	57	7	12.3		Grey Teal	2006 Nov	121	5	4.1	
Duck sp.	2006 Jul	89	4	4.5							
Teal sp.	2007 Aug	141	8	5.7							
Grey Teal	2006 Sep	77	6	7.8							
Duck sp.	2007 Sep	114	14	12.3							
Australian Wood Duck	2007 Sep	16	1	6.3							
Subtyped											
Grey Teal	2005 Nov	203	2		1 H3	Chestnut Teal	2006 Mar	204	18		2 H3
Grey Teal	2005 Dec	44	1		1 H3	Teal sp./Pacific Black Duck	2007 Oct	12	1		1 H3
Duck sp.	2006 Sep	151	2		1 H3	Pacific Black Duck	2008 Mar	94	6		1 H3
Duck sp.	2007 Apr	50	5		4 H3						
Eastern Curlew	2007 May	109	4		2 H3						
Teal sp.	2007 Aug	141	8		2 H3						
Duck sp.	2007 Sep	69	10		4 H3						
Pacific Black Duck	2008 Mar	33	3		1 H3						
Chestnut Teal	2008 Apr	163	6		1 H3						
Pacific Black Duck	2006 Jun	96	2		1 H5	Chestnut Teal/Aust. Shoveler	2006 Mar	217	21		3 H5
Pacific Black Duck	2007 May	236	8		1 H5	Duck sp.	2007 Jan	341	20		2 H5
Duck sp.	2007 Sep	45	4		1 H5	Red-necked Stint	2007 Feb	62	1		1 H5
Duck sp.	2007 Dec	132	6		6 H5	Pacific Black Duck	2008 Mar	94	6		2 H5
Chestnut Teal	2008 Jan	79	2		1 H5						
Duck sp.	2008 Feb	114	5		1 H5						
Grey Teal	2006 Sep	77	6		1 H7	Grey Teal	2006 Nov	121	5		2 H7
Duck sp.	2007 Feb	70	4		3 H7	Duck sp.	2007 Jan	270	14		2 H7
Duck sp.	2007 Apr	146	11		3 H9						
Eastern Curlew	2007 May	109	4		1 H9						

Pacific Black Duck	2007 May	236	8		3 H9						
Duck sp.	2007 May	90	3		1 H9						
Duck sp.	2008 Mar	147	9		2 H9						
Bar-tailed Godwit	2008 Apr	64	4		2 H9						
Far Eastern Curlew	2008 Apr	40	2		2 H9						
Black Swan	2008 Apr	13	1		1 H9						
Chestnut Teal	2008 Ap	163	6		1 H9						

*Increased detection rates were identified as increases above 2.5% in shorebirds and 4.0% in waterfowl. Shorebirds refers to migratory shorebirds only. Note only the numbers and positives from individual sampling events are presented so some numbers differ from those in Table 1 in text. Pos, number of PCR-positives; PPR, percentage PCR-positive rate.
